# On Inflammation of the Cervical Lymphatic Ganglia

**Published:** 1859-12

**Authors:** H. Larrey

**Affiliations:** Member of the Council of Health of the Army, etc.


### TRANSLATIONS.

### ON INFLAMMATION OF THE CERVICAL LYMPHATIC GANGLIA. (*ADENITE CERVICALE.*)

BY H. LARREY, MEMBER OF THE COUNCIL OF HEALTH OF THE ARMY, ETC.

We translate the following summary of a valuable memoir on this subject, read before the Academy of Medicine, Paris, 21st May, 1849, by the author:

"This disease is very frequent in the army, and therefore well known to military surgeons."

"It has been too exclusively regarded by most authors, ancient and modern, as allied to scrofula."

"It is in fact often scrofulous, but is also oftener due to other general causes, such as the influence of atmospheric localities, regimen, habits, particularly such as are inherent in the life of the soldier."

"It is often produced by local causes, such as affections of the scalp, face, ears, mouth, etc., also by mechanical compression of the neck."

"Its seat is in the parotid, sub-maxillary, mastoid, sub-hyoid carotid and supra-claviclar regions."

"It may be acute or chronic, and present variable local characteristics, without necessarily giving rise to general symptoms."

"When uncomplicated, it is generally easily diagnosticated."

"Its course is sometimes rapid, oftener slow, and it often lasts longer than the cause which has produced it."

"It may terminate by resolution, or by suppuration and ulceration, or by induration and degeneration. Its anatomical characters are consequently very variable."

"Its prognosis is often unfavorable, so much so as to require discharge from the military service."

"It requires, according to its causes, forms and terminations, divers means of treatment — preventive, curative, hygienic, medical and surgical."

"It sometimes requires extirpation, from considerable hypertrophy, chronic induration or degeneration, and the operation, although sometimes delicate and difficult, is in these conditions generally followed by favorable results."

